# Decreased Trabecular Bone Mass in *Col22a1*-Deficient Mice

**DOI:** 10.3390/cells10113020

**Published:** 2021-11-04

**Authors:** Wenbo Zhao, Philip Wiedemann, Eva Maria Wölfel, Mona Neven, Stephanie Peters, Thomas Imhof, Manuel Koch, Björn Busse, Michael Amling, Thorsten Schinke, Timur Alexander Yorgan

**Affiliations:** 1 Department of Osteology and Biomechanics, University Medical Center Hamburg-Eppendorf, 20246 Hamburg, Germany; w.zhao.ext@uke.de (W.Z.); philip.wiedemann@stud.uke.uni-hamburg.de (P.W.); e.woelfel@uke.de (E.M.W.); mo.neven@uke.de (M.N.); peters-stephanie@gmx.net (S.P.); b.busse@uke.uni-hamburg.de (B.B.); amling@uke.de (M.A.); 2 Center for Biochemistry, Medical Faculty, University of Cologne, 50923 Cologne, Germany; imhoft@uni-koeln.de (T.I.); Manuel.Koch@uni-koeln.de (M.K.); 3 Medical Faculty, Institute for Dental Research and Oral Musculoskeletal Biology, University of Cologne, 50923 Cologne, Germany

**Keywords:** bone remodeling, *Col22a1*, osteoclasts, osteoblasts

## Abstract

The bone matrix is constantly remodeled by the coordinated activities of bone-forming osteoblasts and bone-resorbing osteoclasts. Whereas type I collagen is the most abundant bone matrix protein, there are several other proteins present, some of them specifically produced by osteoblasts. In a genome-wide expression screening for osteoblast differentiation markers we have previously identified two collagen-encoding genes with unknown function in bone remodeling. Here we show that one of them, *Col22a1*, is predominantly expressed in bone, cultured osteoblasts, but not in osteoclasts. Based on this specific expression pattern we generated a *Col22a1*-deficient mouse model, which was analyzed for skeletal defects by µCT, undecalcified histology and bone-specific histomorphometry. We observed that *Col22a1*-deficient mice display trabecular osteopenia, accompanied by significantly increased osteoclast numbers per bone surface. In contrast, cortical bone parameters, osteoblastogenesis or bone formation were unaffected by the absence of *Col22a1*. Likewise, primary osteoblasts from *Col22a1*-deficient mice did not display a cell-autonomous defect, and they did not show altered expression of Rankl or Opg, two key regulators of osteoclastogenesis. Taken together, we provide the first evidence for a physiological function of *Col22a1* in bone remodeling, although the molecular mechanisms explaining the indirect influence of *Col22a1* deficiency on osteoclasts remain to be identified.

## 1. Introduction

The skeleton is a highly complex tissue, which needs to be constantly remodeled in order to provide life-long stability. This remodeling process is mediated by two fundamentally different cell types, i.e., bone-forming osteoblasts and bone-resorbing osteoclasts [1,2]. Whereas multinucleated osteoclasts are generated by fusion of hematopoietic progenitor cells in a process regulated by the cytokine Rankl and its antagonist Opg, osteoblasts derive from mesenchymal progenitor cells to accumulate in cellular groups producing an extracellular matrix that subsequently mineralizes [3,4]. In this process hydroxyapatite, i.e., crystalline calcium phosphate, is incorporated into a scaffold of collagen fibrils, which mostly consist of type I collagen, the most abundant protein of the bone matrix [5]. The importance of proper synthesis, processing and folding of type I collagen for skeletal integrity is underscored by the fact that mutations in the genes encoding either the type I collagen subunits or enzymes required for its posttranslational modification, cause osteogenesis imperfecta, a severe skeletal disorder with poor bone quality and high fracture risk [6]. Furthermore, type I collagen is also a main constituent of dentin, produced by odontoblasts, which explains why osteogenesis imperfecta can be associated with dental defects [7].

Besides type I collagen there are many other less abundant bone matrix proteins, some of which are specifically produced by osteoblasts and/or odontoblasts. The majority of these proteins have been analyzed for a potential role in regulating bone matrix integrity and turnover, which also included the generation and analysis of mouse deficiency models [8]. Here it was found that specific proteins are required to ensure the unique material properties of the bone matrix, whereas others additionally served initially unexpected functions [9,10,11]. One example for the latter is Dmp1, an extracellular matrix protein specifically produced by terminally differentiated osteoblasts and odontoblasts. It was found that Dmp1-deficient mice display severe defects of bone matrix mineralization, yet this phenotype was primarily explained by hypophosphatemia, as a consequence of increased bone-specific production of the phosphaturic hormone Fgf23 [12,13]. A similar pathology is caused by deficiency of another gene with specific expression in terminally differentiated osteoblasts and odontoblasts, i.e., *Phex*, encoding a transmembrane endopeptidase [14]. Moreover, the identification of inactivating *DMP1/PHEX* or activating *FGF23* mutations in patients with hypophosphatemic rickets has underscored the human relevance of these genes, all of them displaying a skeleton-specific expression pattern [15].

Therefore, based on the hypothesis that osteoblast-specific expression potentially predicts a physiological function in the skeleton, we have previously performed a screen for novel osteoblast differentiation markers [16]. More specifically, we applied genome-wide expression analysis to identify genes with increasing expression during the course of primary osteoblast differentiation. For the hundred genes with the strongest induction, we applied RT-PCR to compare the expression in bone (femur, calvaria) and seven non-skeletal tissues. We hereby confirmed the bone-specific expression of various established genes, including *Dmp1* and *Phex*, but we also identified other genes with a similar expression pattern. While we have previously analyzed the putative function of one of these genes, i.e., *Panx3*, the present study is focused on *Col22a1*, one of two collagen-encoding genes that were identified in our screening approach [16]. Type XXII collagen (COLXXII), the protein encoded by *Col22a1*, was originally identified as a novel tissue junction component, particularly at the myotendinous junction of heart and skeletal muscle [17]. It belongs to the FACIT (fibril-associated collagens with interrupted triple helix) family of collagens, which do not form fibrils [18]. Importantly, however, the physiological functions of COLXXII are essentially unknown, which is also explained by the lack of phenotypic data in a corresponding mouse deficiency model.

Here we show that *Col22a1* is predominantly expressed by osteoblasts, but not by osteoclasts. We further generated a *Col22a1*-deficient mouse model, where we identified a low bone mass phenotype, restricted to the trabecular bone compartment. Histomorphometric quantification revealed that this osteopenia was associated with increased numbers of osteoclasts, whereas bone formation and matrix mineralization were unaffected. Taken together, our findings provide the first evidence for a specific role of *Col22a1* in regulating skeletal turnover.

## 2. Materials and Methods

### 2.1. Ex Vivo Cultures

Primary murine calvarial osteoblasts were isolated by sequential collagenase digestion from the calvariae of 3–5 days old mice as previously described [16]. Primary bone marrow osteoblasts were generated by plating full bone marrow from 12–15-week-old mice at a density of 5 × 10^6^ cells/mL. Cells were cultured in α-MEM supplemented with 10% FBS and 100 U/mL penicillin/streptomycin until they reached 8% confluency (day 0). We then added ascorbic acid (50 µg/mL) and β-glycerophosphate (10 mM) to the cultures to induce osteogenic differentiation. Alizarin red staining and quantification was performed as previously described [19].

Primary osteoclast cultures were generated from full bone marrow of 12–15-week-old mice plated at a density of 5 × 10^6^ cells/mL. Cells were cultured in α-MEM supplemented with 10% FBS and 100 U/mL penicillin/streptomycin. Differentiation was facilitated by addition of 10 nM Vitamin D_3_ (Sigma-Aldrich) for the first 5 days of culture and the supplementation with 10 nM Vitamin D_3_, 20 ng/mL MCSF, and 40 ng/mL msRANKL (both Peprotech) for the remaining duration of the culture. Trap staining was performed as previously described [20].

### 2.2. Expression Analysis

RNA from murine tissues (of 10-week-old C57Bl/6J mice), cultured calvarial osteoblasts, or bone-marrow-derived osteoblasts/osteoclasts was isolated using the Macherey Nagel NucleoSpin kit (Macherey-Nagel GmbH & Co. KG, Düren, Germany). Concentration and quality of RNA were measured using a NanoDrop ND-1000 system (NanoDrop Technology, Wilmington, DE, USA). For qRT-PCR expression analysis 500 ng of RNA was reverse transcribed using Verso cDNA Kit (Thermo Fisher Scientific Inc., Waltham, MA, USA) according to manufacturer’s instructions. The quantitative expression analysis was performed using a StepOnePlus system and predesigned TaqMan gene expression assays (*Col1a1* Mm00801666_g1; *Col13a1* Mm00483507_m1; *Col22a1* Mm01195056_m1; *Runx2* Mm00501580_m1; *Sp7* Mm00504574_m1; *Alpl* Mm00475834_m1; *Ibsp* Mm00492555_m1; *Tnfsf11* Mm00437135_m1; *Tnfrsf11b* Mm00435454_m1; Thermo Fisher Scientific Inc., USA). Gapdh expression was used as an internal control. Relative quantification was performed according to the ΔΔCT method. Alternatively, quantification relative to Gapdh was applied for individual samples. The applicable method is stated in the figure legends.

### 2.3. Animal Husbandry and Experiments

Col22a1-deficient mice (C57BL/6N-*Col22a1*^em1Rbrc^) were commercially obtained as frozen sperms from RIKEN BRC, Japan (RBRC06370) and revitalized in our animal facility according to standard protocols. In this mouse line 37 nucleotides of exon 15 had been deleted via CRISPR/Cas9-mediated endonuclease activity, leading to a premature stop codon. Genotyping was performed using specific forward primers for the wildtype (WT) (5′-GACCTCCTGGACTACCAGGGCC-3′) and knockout (KO) allele (5′-TGTGGACTCTCTTCTGCAGGAC-3′) together with a common reverse primer (5′-AGCAGTGACATGCCTGCACGTG-3′) resulting in a 463 bp WT and 455 bp KO PCR product. All mice were kept in a specific pathogen-free environment with a 12-h light/dark cycle, 45–65% relative humidity, and 20–24 °C ambient temperature in open or individually ventilated cages with wood shavings bedding and nesting material in groups not surpassing 6 animals. The mice had access to tap water and standard rodent chow (1328P, Altromin Spezialfutter GmbH & Co. KG, Lage, Germany) ad libitum. For determination of the bone formation rate, the mice received two i.p. injections of calcein (50 mg/kg) 9 and 2 days prior to sacrifice. The number of animals per group was 5–6, with the exact number represented by the individual data points in the corresponding figures. All animal experiments were approved by the animal facility of the University Medical Center Hamburg-Eppendorf and by the “Behörde für Soziales, Familie, Gesundheit und Verbraucherschutz” (Org869, G73/18) in accordance with the local implementation of EU Directive 2010/63/EU for animal experiments.

### 2.4. Radiological Assessment

The animals were sacrificed via CO_2_ intoxication and body mass and length (base of the skull to root of the tail) were recorded. The skeletons were fixed in 3.7% PBS-buffered formaldehyde for 48 h prior to storage in 80% ethanol. µCT scanning and microarchitectural analysis were performed with a voxel resolution of 10 µm (1000 projections per slice with 2048 samples and 200 s sample time at 55 kVp, 145 µA) using a μCT 40 desktop cone-beam microCT (Scanco Medical AG, Wangen-Brüttisellen, Switzerland) as previously described [21] according to standard guidelines [22]. In detail, trabecular bone was analyzed in the distal metaphysis of the femur in a volume situated 2500 μm to 500 μm proximal of the distal growth plate with evaluation script “UCT_EVALUATIONV6_MULTIAUTO” of the Scanco MicroCT software suite (v6.5-2, Scanco Medical, Switzerland) using the following parameters: Seg: 0.8|1|250|1000|6|0.8|1|250|1000|6|0.8|2| 300|1000|6; Misc: 25|30|10|2|3|1). Cortical bone was analyzed in a volume with a length of 1000 µm situated in the middle of the femoral diaphysis with the same integrated evaluation script as above with these parameters: Seg: 0.8|1|300|1000|6|0.8|1|300|1000|6|0.8|2|350|1000|6|0.8|2|350|1000|6; Misc: 10|20|1|1|0|1.

### 2.5. Skeletal Histology

For bone histology, the fixed lumbar vertebral bodies L1 to L4 were dehydrated in ascending alcohol concentrations and then embedded in methylmetacrylate as described previously [23]. Sections of 4 μm or 12 µm thickness were cut in the sagittal plane on a Microtec rotation microtome (Techno-Med GmbH, Bielefeld, Germany). These were stained by the von Kossa/van Gieson or Toluidine blue staining procedure as previously described [24]. Histomorphometry was performed according to the ASBMR guidelines [25] using the Bioquant Osteo histomorphometry system (BIOQUANT Image Analysis Corp., Nashville, TN, USA) for trabecular bone microarchitecture analysis or the Osteomeasure system (OsteoMetrics, Inc., Decatur, GA, USA) for cellular and dynamic histomorphometry.

### 2.6. Biomechanical Testing

For biomechanical testing, a three-point bending test was performed on explanted femora using a universal testing machine Z2.5/TN1S and the testXpert software (both Zwick Roell, Ulm, Germany). Femora were horizontally mounted and centrally positioned with the posterior surface facing downward at a support distance of 7 mm. A constant displacement rate of 0.05 mm/s was applied. Load-displacement data were recorded until failure. Results were calculated in the testXpert software.

### 2.7. Biochemical Assays

Blood was collected from mice at sacrifice via intracardial aspiration. After coagulation at room temperature for 30 min, serum was separated by centrifugation (6 min, 3300× *g*) and stored at −80 °C until further use. Serum concentrations of Rankl (MTR00, R&D Systems, USA) and Osteoprotegerin (MOP00, R&D Systems, Minneapolis, MN, USA) were measured by ELISA according to the manufacturer’s instructions.

### 2.8. Statistical Analysis

All data presented in the manuscript are presented as individual values and as means ± standard deviations. Statistical analysis was performed via unpaired, two-tailed Student’s *t*-test with Bonferroni correction for multiple testing using Prism software (GraphPad Software, San Diego, CA, USA). *p*-values below 0.05 were considered statistically significant. Sample size and power of analysis for animal experiments was determined by an a priori *t*-test with G*Power [26].

## 3. Results

### 3.1. Col22a1 Is Predominantly Expressed in Bone-Forming Osteoblasts

We have previously applied an unbiased screening approach with the aim to identify molecules with specific expression in bone-forming osteoblasts [16]. For that purpose, we first performed Affymetrix Gene hybridization to sort all genes according to the highest level of induction from day 5 to day 12 of primary calvarial osteoblast differentiation. Thereafter, we analyzed the expression of the 100 most strongly induced genes by semi-quantitative RT-PCR in 7 non-skeletal tissues, compared to femur and calvaria. Here we observed predominant bone expression for known osteoblast markers (*Ibsp, Dmp1, Phex, Bglap*, etc.), but also for some other genes, including two collagen-encoding genes, such as, *Col13a1* and *Col22a1* [16]. In the present study we have expanded our expression analysis and applied qRT-PCR in an extended panel that included 16 different tissues.

**Figure 1:**
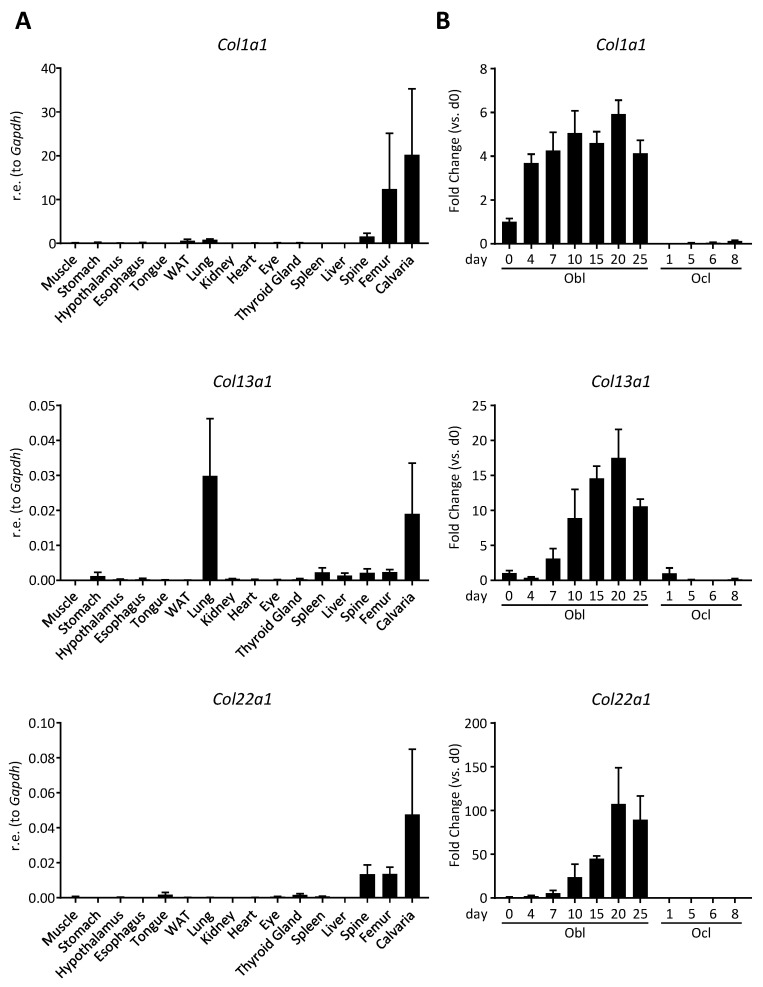
Specificity of *Col22a1* expression. (**A**) qRT-PCR-based expression analysis of *Col1a1*, *Col13a1*, and *Col22a1* relative to *Gapdh* in different tissues as indicated; (**B**) qRT-PCR-based expression analysis of *Col1a1*, *Col13a1,* and *Col22a1* in osteoblasts and osteoclasts during ex vivo differentiation at the indicated timepoints (relative to osteoblasts at day 0). *n* = 3 independent samples per group. Error bars indicate standard deviation.

Here we analyzed the expression pattern of *Col13a1* and *Col22a1* in comparison to *Col1a1*, the latter encoding the alpha subunit of type I collagen (Figure 1A). For *Col13a1*, we detected robust expression not only in bone (spine, femur calvaria), but also in other tissues, especially in the lung. In stark contrast, *Col22a1* expression displayed a remarkable specificity for bone tissue, similar to the expression pattern of *Col1a1*. We additionally monitored the expression of the three genes throughout ex vivo differentiation of primary calvarial osteoblasts or bone marrow osteoclast cultures (Figure 1B). As expected, based on the data from our previous study [16], the expression of all three analyzed collagen genes was strongly increasing during the course of primary osteoblast differentiation, whereas expression in osteoclast cultures was barely detectable for all three collagen-encoding genes. Taken together, these data identify *Col22a1* as a novel osteoblast differentiation marker with predominant expression in bone.

### 3.2. Generation of Col22a1-Deficient Mice

**Figure 2:**
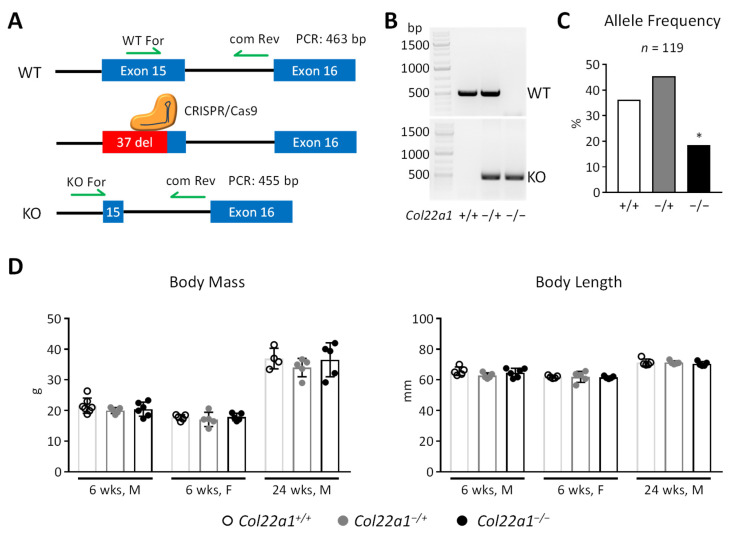
Generation of *Col22a1*-deficient mouse model. (**A**) Schematic overview of the genetic modifications leading to *Col22a1* loss-of-function and binding sites of genotyping primers. (**B**) Representative image of an agarose gel electrophoresis (ethidiumbromide stain) with PCR products used for genotyping of the *Col22a1*-deficiency mouse model. (**C**) Allele Frequency chart for 119 offspring mice from heterozygous matings. Data were compared to the expected Mendelian ratio by chi-squared test. (**D**) Body mass and body length of 6- and 24-week-old male and female *Col22a1*^+/+^, *Col22a1^−^*^/+^ and *Col22a1*^−/−^ mice as indicated. Single points indicate individual animals. Error bars indicate standard deviation. Data were analyzed by Student’s *t*-test with Bonferroni correction for multiple testing. * *p* < 0.05.

Given the remarkably specific expression pattern identified for *Col22a1*, we hypothesized that the presence of COLXXII is important for skeletal integrity. Since the physiological functions of *Col22a1* have not yet been studied by phenotypic analysis of a corresponding mouse deficiency model, we took advantage of a commercially available *Col22a1*-deficiency model. This line was generated via CRISPR/Cas9 gene editing to remove 37 bp from exon 15 of the *Col22a1* gene, which is predicted to cause a loss-of-function, since the coding region of the *Col22a1* transcript is covered by 63 exons (Figure 2A). Starting from frozen sperms we established a colony in our animal facility and were able to generate heterozygous (*Col22a1*^−/+^) and homozygous (*Col22a1*^−/−^) animals (Figure 2B), although the latter were born slightly below the expected Mendelian frequency (Figure 2C). Otherwise, we did not observe any obvious abnormalities caused by *Col22a1*-deficiency, which led us to study the phenotype of wildtype, heterozygous and homozygous littermate animals at 6 weeks (male and female) and 24 weeks (male) of age. In all analyzed groups, the body mass and length of *Col22a1*^−/+^ and *Col22a1*^−/−^ mice did not differ significantly from their wildtype controls (Figure 2D).

### 3.3. Moderate Trabecular Osteopenia, but Normal Cortical Bone Mass in Col22a1-Deficient Mice

**Figure 3:**
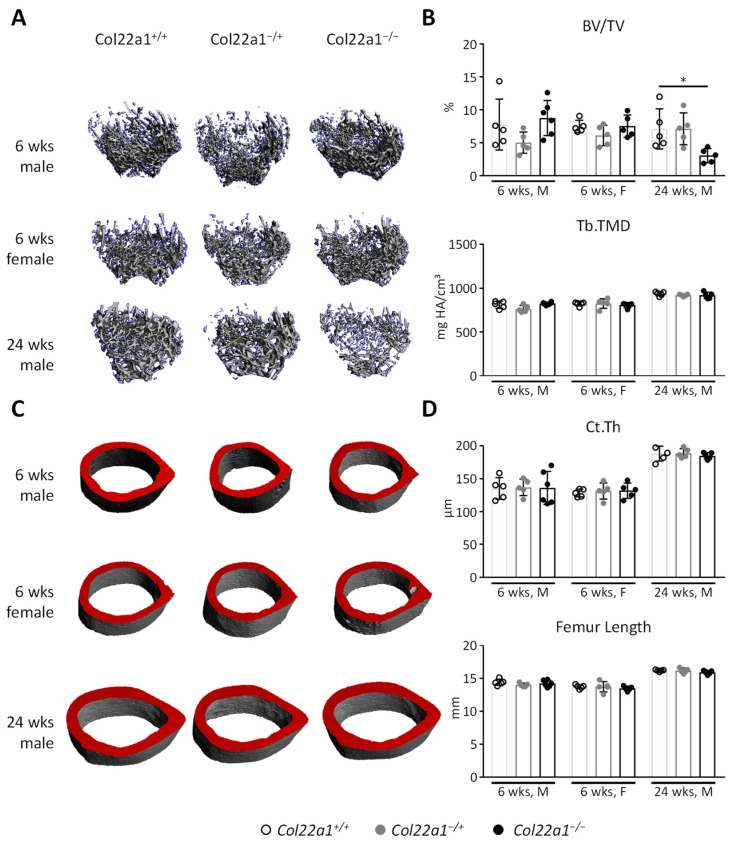
Femoral phenotype of *Col22a1*-deficient mice. (**A**) Representative µCT reconstructions of the distal metaphyseal trabecular bone from femora of mice with the indicated age, gender, and genotype. (**B**) µCT-based quantification of the bone volume/tissue volume (BV/TV) and trabecular tissue mineral density (Tb.TMD) in 6- and 24-week-old male and female *Col22a1*^+/+^, *Col22a1^−^*^/+^, and *Col22a1*^−/−^ mice as indicated. (**C**) Representative µCT reconstructions of the mid-diaphyseal cortical bone from the femora of mice with the indicated age, gender, and genotype. The virtual cutplanes appear red to highlight the wall thickness. (**D**) µCT-based quantification of the cortical thickness (Ct.Th) and femur length in the same mice. Single points indicate individual animals. Error bars indicate standard deviation. Data were analyzed by Student’s *t*-test with Bonferroni correction for multiple testing. * *p* < 0.05.

Initial investigation of the bone microarchitecture via µCT analysis of the femur indicated a significant reduction of trabecular bone mass (BV/TV) as well as reduced trabecular bone mineral density and content in 24-week-old male *Col22a1*^−/−^ mice (Figure 3A,B and Figure S1). In younger mice, at the age of 6 weeks, we did not observe changes in femoral trabecular bone mass in both male and female mice. Furthermore, trabecular tissue mineral content, cortical thickness, and femur length as well as cortical area, bone mineral density, and content were not affected by *Col22a1*-deficiency in any of the analyzed groups (Figure 3B–D and Figure S1).

**Figure 4:**
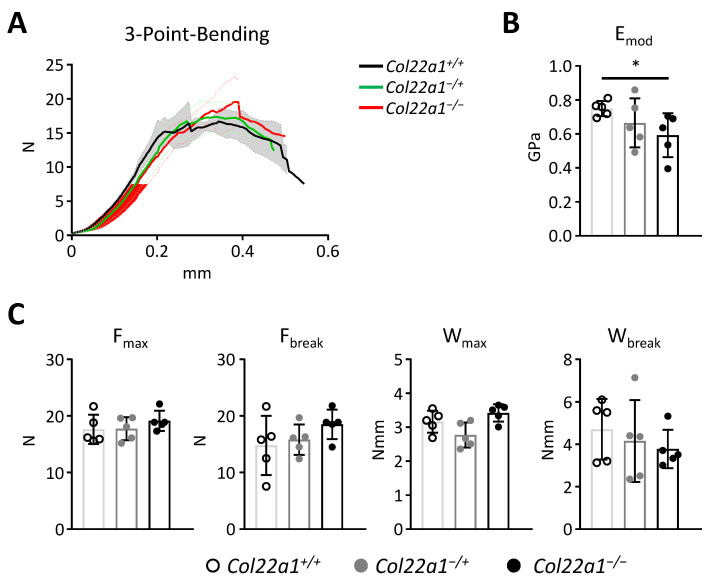
Mechanical properties of femora from *Col22a1*-deficient mice. (**A**) Load displacement curve of the three-point bending assays performed on the explanted femora of 24-week-old male *Col22a1*^+/+^, *Col22a1^−^*^/+^, and *Col22a1*^−/−^ mice. Solid lines represent the mean value, the semitransparent areas denote the standard deviation. (**B**) Material stiffness (E_mod_) as determined by the three-point bending test. (**C**) Maximum load (F_max_), load at the breaking point (F_break_), work required to reach the maximum load point (W_max_) and work required to reach the breaking point (W_break_) as determined by the three-point bending test. Single points indicate individual animals. Error bars indicate standard deviation. Data were analyzed by Student’s *t*-test with Bonferroni correction for multiple testing. * *p* < 0.05.

Mechanical testing of the femora from 24-week-old male *Col22a1*^−/+^ and *Col22a1*^−/−^ mice as well as wildtype littermate controls indicated moderately altered properties of the bone matrix of *Col22a1*^−/−^ mice, based on a significantly reduced matrix stiffness (Figure 4A,B). However, this reduction of the elastic modulus did not translate into significantly different mechanical properties with regard to maximum load and fracture resistance (Figure 4C).

### 3.4. Increased Osteoclast Numbers at Trabecular Bone Surfaces of Col22a1-Deficient Mice

**Figure 5:**
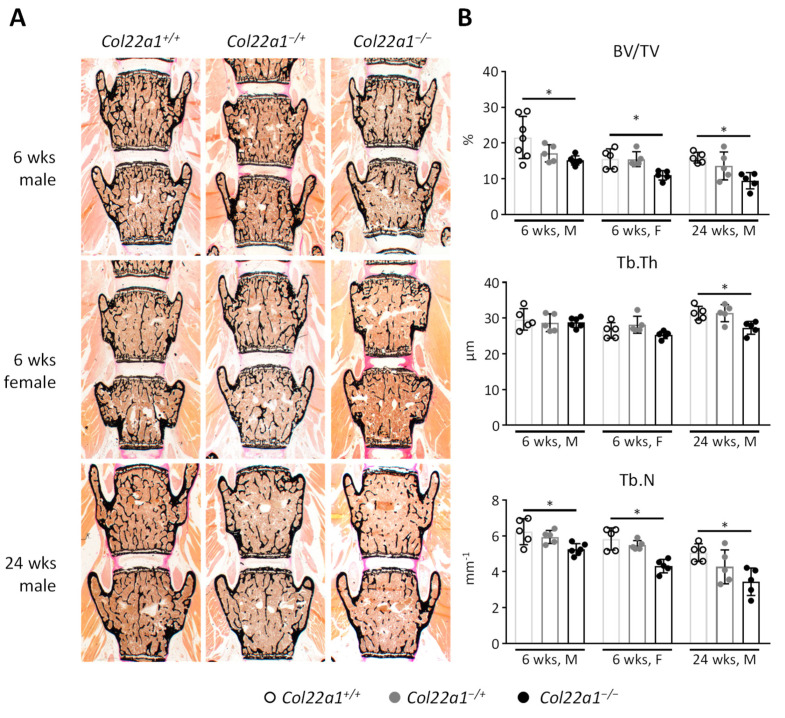
Structural histomorphometry of *Col22a1*-deficient vertebral bodies. (**A**) Representative images of the vertebral bodies L3-L4 (van Gieson/von Kossa stain) of mice with the indicated age, gender, and genotype. (**B**) Histomorphometric quantification of the bone volume/tissue volume (BV/TV), trabecular thickness (Tb.Th), and trabecular number (Tb.N) in the vertebral bodies of the indicated animals. Single points indicate individual animals. Error bars indicate standard deviation. Data were analyzed by Student’s *t*-test with Bonferroni correction for multiple testing. * *p* < 0.05.

In-depth phenotyping by undecalcified histology of the vertebral bodies from *Col22a1*^+/+^, *Col22a1*^−/+^, and *Col22a1*^−/−^ mice revealed a more severe phenotype with significant reduction of the trabecular bone volume (BV/TV) in *Col22a1*-deficient mice that was already apparent in male and female mice at the age of 6 weeks (Figure 5A,B). Closer analysis of the microarchitectural properties of the trabecular bone compartment highlighted that this decrease in trabecular bone mass was mostly attributed to a significant reduction of the trabecular number that was consistently observed in *Col22a1*-deficent mice throughout all groups. Additionally, 24-week-old *Col22a1*^−/−^ mice also displayed a significant reduction in trabecular thickness. It is also important to state that we did not observe enrichment of non-mineralized bone matrix, i.e., osteoid, in *Col22a1*^−/−^ mice.

**Figure 6:**
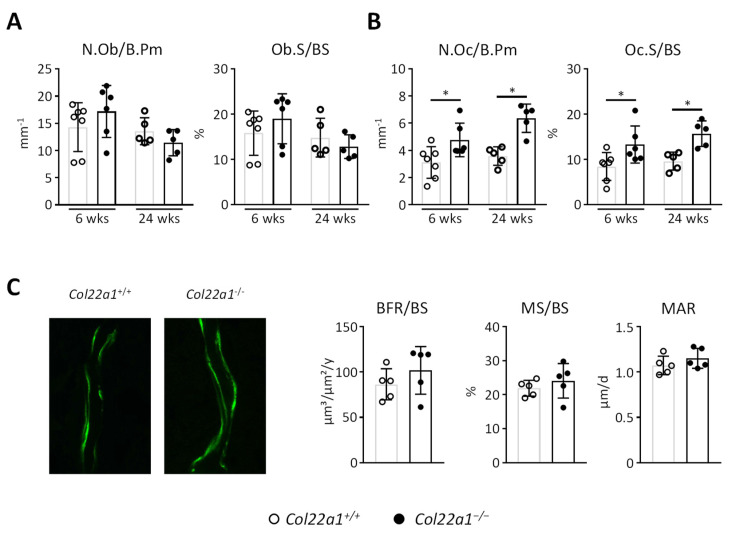
Cellular and dynamic histomorphometry of *Col22a1*-deficient vertebral bodies. (**A**) Number of osteoblasts per bone perimeter (N.Ob/B.Pm) and osteoblast surface per bone surface (Ob.S/BS) as determined in the trabecular compartment of the vertebral bodies from 6- and 24-week-old, male *Col22a1*^+/+^ and *Col22a1*^−/−^ mice. (**B**) Number of osteoclasts per bone perimeter (N.Oc/B.Pm) and osteoclast surface per bone surface (Oc.S/BS) as determined in the same sections. (**C**) Representative images of calcein labels on the vertebral trabecular bone of 24-week-old, male *Col22a1*^+/+^ and *Col22a1*^−/−^ mice. Bone formation rate per bone surface (BFR/BS), mineralizing surface per bone surface (MS/BS) and mineral apposition rate (MAR) as determined in the trabecular compartment of the vertebral bodies of these mice. Single points indicate individual animals. Error bars indicate standard deviation. Data were analyzed by Student’s *t*-test. * *p* < 0.05.

Next, we applied cellular and dynamic histomorphometry to define the underlying cellular cause for the observed trabecular osteopenia in *Col22a1*-deficient mice. Here we did not observe a significant difference toward wildtype littermates for the number of osteoblasts per bone perimeter or the osteoblast surface per bone surface (Figure 6A). In contrast, we unexpectedly identified a significant increase in the osteoclast number and surface in 6- and 24-week-old male *Col22a1*^−/−^ mice (Figure 6B). Furthermore, dynamic histomorphometry performed in the 24-week-old male mice revealed that osteoblast activity, as determined by quantification of the bone formation rate, mineralizing surface and mineral apposition rate, was not altered in the *Col22a1*^−/−^ mice (Figure 6C).

### 3.5. Primary Osteoblasts from Col22a1-Deficient Mice Do Not Display a Cell-Autonomous Osteogenesis Defect

**Figure 7:**
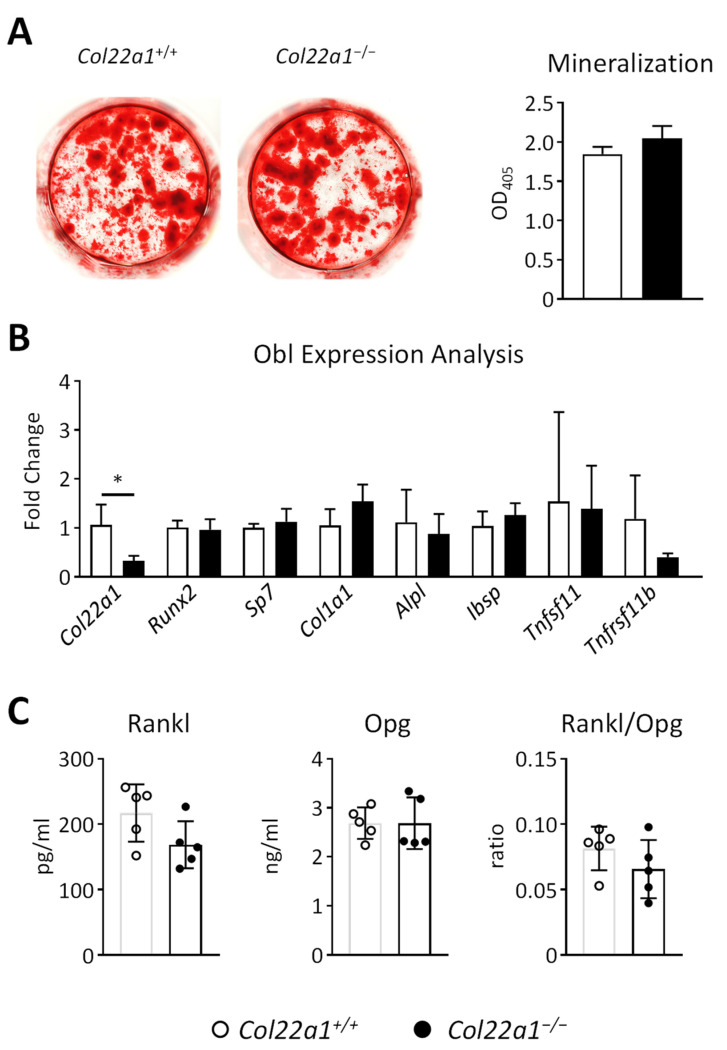
Ex vivo analysis of *Col22a1*-deficient osteoblasts. (**A**) Representative images and quantification of alizarin red staining of primary bone marrow cells isolated from *Col22a1*^+/+^ and *Col22a1*^−/−^ mice after 10 days of osteogenic differentiation. (**B**) qRT-PCR-based expression analysis of the indicated genes in *Col22a1*^−/−^ bone marrow cells after 10 days of osteogenic differentiation relative to data obtained from *Col22a1*^+/+^ cells. (**C**) Concentration of Rank ligand (Rankl) and Osteoprotegerin (Opg) as well as the Rankl/Opg ratio as determined by ELISA in the serum of 24-week-old male *Col22a1*^+/+^ and *Col22a1*^−/−^ mice. *n* = 4 or as indicated by individual symbols independent samples per group. Error bars indicate standard deviation. Data were analyzed by Student’s *t*-test. * *p* < 0.05.

Given the high expression of *Col22a1* during the course of primary osteoblast differentiation, we next isolated bone marrow cells from wildtype and *Col22a1*-deficient mice and induced their osteogenic differentiation by addition of ascorbic acid and β-glycerophosphate. By quantification of matrix mineralization via alizarin red staining we did not observe a genotype-dependent difference after 10 days of osteogenic differentiation (Figure 7A). This was also supported by qRT-PCR expression analyses, where the only gene with differential expression was *Col22a1* itself (Figure 7B). Moreover, the expression of the most relevant osteoclastogenesis regulators, i.e., Rankl and Opg, encoded by *Tnfsf11* and *Tnfrsf11b*, respectively, was not significantly altered. Consistently, the serum levels of Rankl and Opg, as well as the critical ratio of Rankl to Opg, were not different in *Col22a1*-deficient mice compared to wildtype littermates (Figure 7C). Therefore, the molecular mechanism explaining the indirect influence of *Col22a1* deficiency on osteoclast numbers remains to be elucidated.

## 4. Discussion

Bone remodeling is mediated by bone-resorbing osteoclasts and bone-forming osteoblasts, two entirely different cell populations. While osteoid, the organic component of the bone matrix, consists mostly of type I collagen that provides the scaffold for the mineral phase consisting of calcium phosphate crystals, there are numerous other proteins that contribute to the composition of the bone matrix and are essential for its proper function [8]. In a previous study we have analyzed the transcriptome of differentiating osteoblasts and observed many well-established osteoblastogenesis markers among the genes with the strongest induction during differentiation, thereby demonstrating the validity of our approach. In this screening process, we also identified several genes with a hitherto unknown function in skeletal turnover, despite predominant expression in bone tissue. While the main objective of our previous study was related to *Panx3* [16], our current investigation is focused on the role of *Col22a1*, encoding type XXII collagen (COLXXII). *Col22a1* was one of two collagen transcripts identified among the hundred most strongly induced genes during osteoblast differentiation. In the current study we were able to show that *Col22a1*, unlike *Col13a1*, is predominantly expressed in bone tissue and differentiated osteoblasts, which led us to investigate its putative role in the skeleton.

COLXXII has originally been identified as a novel tissue junction component, as it is mostly localized in the transition zones of tissues, such as the myotendinous junction of heart and skeletal muscle, the articular cartilage/synovial fluid junction, and the junction of hair follicles and the dermis [17]. Targeting of *Col22a1* in Zebrafish morpholino models has led to the observation that these animals display muscular dystrophy most likely due to a severely disrupted myotendinous junction that could even lead to muscle detachment [27]. In a different Zebrafish study, genetic disruption of the *Col22a1* gene has led to an increased risk of intracranial hemorrhages due to increased vascular permeability [28]. The latter was attributed to a disruption of the proper structure of the basal lamina. These findings were supported by the association of genetic variants of human *COL22A1* with a higher risk of aneurysms [28,29]. Importantly, however, since there is no published analysis on the phenotype of a *Col22a1*-deficient mouse model, the physiological role of COL XXII is still not well-defined.

Based on the observed *Col22a1* expression pattern, our primary focus was to generate such a mouse model in order to study their skeletal phenotype. Importantly, we did not observe any obvious morphological alterations or extraskeletal phenotypes, such as aneurysms. However, we did observe significantly less *Col22a1*-deficient mice born than would be expected from the Mendelian ratio. Nonetheless, it remains purely speculative at this point, whether this discrepancy can be attributed to one of the previously proposed functions of COLXXII. With respect to the skeleton, it was also obvious that *Col22a1* deficiency did not impair developmental processes, i.e., desmal or endochondral ossification, or skeletal growth. Moreover, we did not detect enrichment of non-mineralized bone, i.e., osteoid, unlike it is the case in mice carrying type I collagen mutations, or in mice lacking Dmp1 [13,30,31]. Finally, there was no indication of impaired osteoblast differentiation and function in association with *Col22a1* deficiency. On the other hand, trabecular osteopenia in vertebral bodies, albeit moderate, was observed in all analyzed groups, where wildtype and heterozygous littermates were used as controls.

Of note, we also observed a significant reduction of trabecular bone mass in the distal femoral metaphysis, yet this difference was only found in the 24-week-old mice. Although this may indicate that the axial skeleton is more affected than the appendicular skeleton in *Col22a1*-deficient mice, there is also another possible explanation for this apparent discrepancy. In fact, since there are far less trabecular bone structures in long bones when compared to the spine, a reduction of trabecular bone mass is generally more robust, when spine sections are analyzed. On the other hand, assessing bone parameters in femora by µCT is advantageous for quantification of cortical parameters in the midshaft region. Here we observed that cortical bone mass and most cortical bone mechanical properties, as determined by a three point bending test, remained unaffected by the loss of *Col22a1*. This indicates that COLXXII is predominantly relevant in the trabecular bone compartment. The only effect on cortical bone that we were able to determine was reduced bone matrix stiffness. It is conceivable to speculate that this phenotype is caused by reduced type I collagen crosslinking via COLXXII, although such a function has not yet been described for COLXXII [32]. Indeed, COLXXII belongs to the FACIT subfamily of collagens, which does not form fibrils but copolymerize into superstructures with fibril-forming collagens and mediate interactions with the environment [33].

When investigating the underlying cause for the reduced trabecular bone mass, we specifically observed increased osteoclast parameters. Since *Col22a1* was found barely expressed in osteoclasts, these results were fully unexpected and suggest that they are indirectly mediated. Since osteoblast lineage cells are major producers of Rankl and Opg, which in turn regulate osteoclastogenesis [34], we particularly analyzed the possibility, that *Col22a1*-deficient osteoblasts produce more Rankl and/or less Opg. By comparing primary osteoblasts isolated from wildtype and *Col22a1*-deficient littermate mice, we did not observe a cell-autonomous osteogenesis defect, and there was no differential expression of the genes encoding Rankl or Opg. Moreover, the serum concentrations of these two factors were not different between wildtype and *Col22a1*-deficient littermate mice. Interestingly, it has been shown that *Col22a1* colocalizes with integrins such as α2β1 and α11β1 in a pattern that is reminiscent of integrin signaling [32]. Moreover, an involvement of *Col22a1* in integrin-mediated mechanosensing and negative regulation of chondrocyte hypertrophy has been hypothesized [32,35]. Therefore, it is conceivable that an altered bone matrix composition lacking COLXXII influences osteoclast differentiation or attachment via integrin signaling [36,37,38]. Further research is required to adequately test these hypotheses and to fully elucidate the underlying mechanism.

Apart from the fact that our current data do not provide a molecular mechanism explaining the increased trabecular osteoclast numbers in *Col22a1*-deficient mice, there are other limitations of our study. At the age of 24 weeks, we only analyzed male mice. Considering the data obtained in 6-week-old animals, it is likely that female mice might display a similar phenotype. However, this has not been verified. Furthermore, since we only analyzed a global knockout model, we cannot exclude that the observed phenotype might be caused by effects originating from other cell types. Therefore, even if we found *Col22a1* to be predominantly expressed by osteoblasts, we truly believe that models with cell-specific deletion of *Col22a1* are required to fully investigate the effect of COLXXII on the skeleton.

## 5. Conclusions

In conclusion, we present the first data on the skeletal phenotype of a Col22a1-deficient mouse model. Our data demonstrate that Col22a1-deficiency specifically causes trabecular osteopenia with an increased osteoclast number, which is not explained by a shifted Rankl/Opg ratio.

## Data Availability

All relevant data are presented in the manuscript; raw data are available upon request from the corresponding authors.

## Supplementary Materials

The following are available online at https://www.mdpi.com/article/10.3390/cells10113020/s1, Figure S1: Extended femoral µCT results.
