# Supplementary material for: Decreased Trabecular Bone Mass in Col22a1-Deficient Mice
Source: Cells. 2021 Nov 4;10(11):3020. doi: 10.3390/cells10113020 (PMC8616175; doi:10.3390/cells10113020)
Supplement: Supplementary file 1 [file cells-10-03020-s001.zip › cells-1423268-supplementary.pdf]

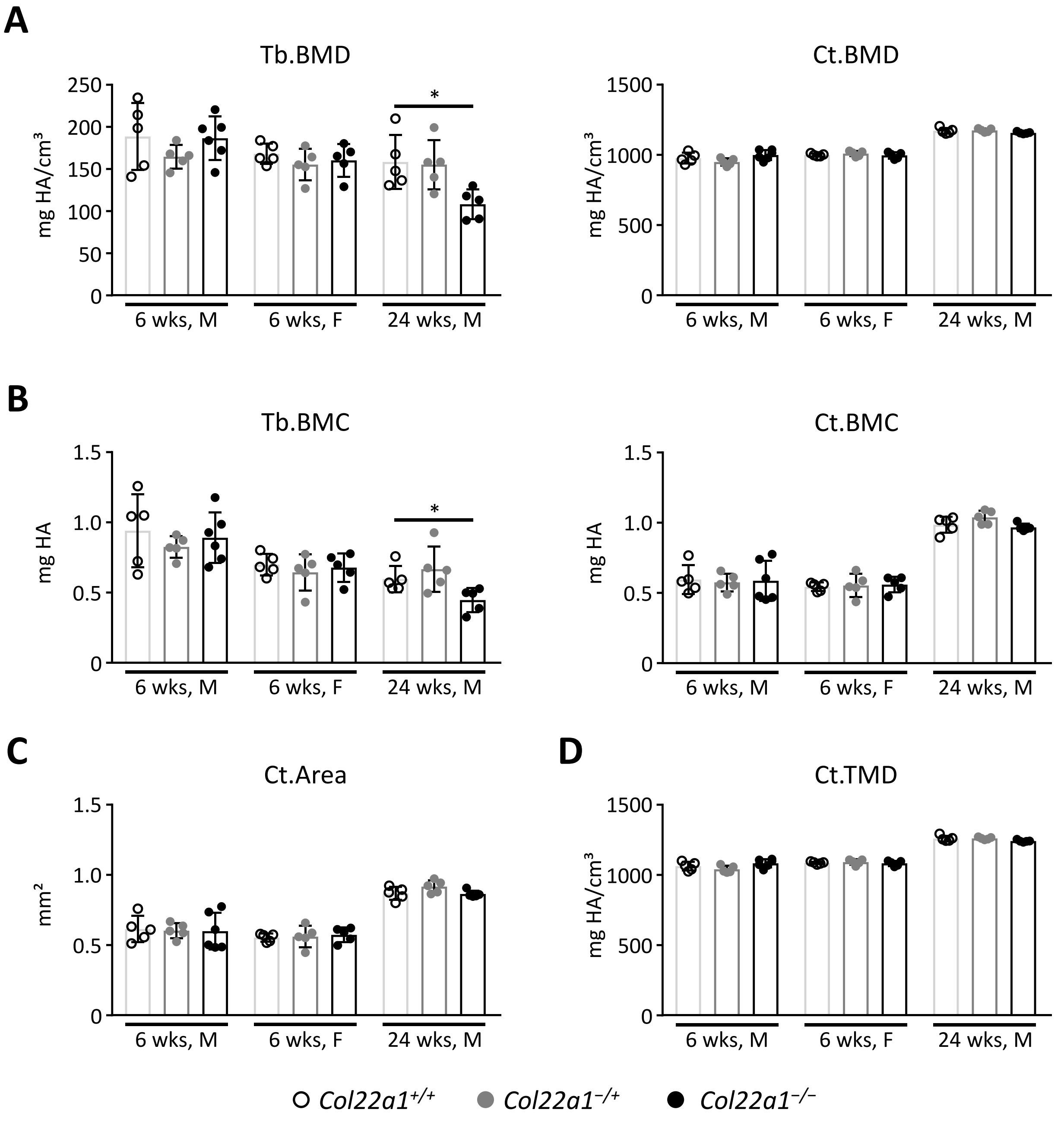

Figure S1: Extended femoral μCT results. **(A)** μCT-based quantification of the trabecular and cortical bone mineral density (Tb.BMD, Ct.BMD) in 6- and 24-week-old male and female *Col22a1*$^{+/+}$, *Col22a1*$^{-/+}$ and *Col22a1*$^{-/-}$ mice as indicated. **(B)** μCT-based quantification the trabecular and cortical bone mineral content (Tb.BMC, Ct.BMC) in the same mice. **(C)** μCT-based quantification cortical area (Ct.Area) in the same mice. **(D)** μCT-based quantification the cortical tissue mineral content (Ct.TMD) in the same mice. Single points indicate individual animals. Error bars indicate standard deviation. Data were analyzed by Student's t-test with Bonferroni correction for multiple testing. *$p < 0.05$.
